# Undecanoic Acid, Lauric Acid, and N-Tridecanoic Acid Inhibit *Escherichia coli* Persistence and Biofilm Formation

**DOI:** 10.4014/jmb.2008.08027

**Published:** 2020-10-13

**Authors:** Xing Jin, Jiacheng Zhou, Gabriella Richey, Mengya Wang, Sung Min Choi Hong, Seok Hoon Hong

**Affiliations:** Department of Chemical and Biological Engineering, Illinois Institute of Technology, Chicago, IL 60616, USA

**Keywords:** *E. coli*, persister cells, biofilms, medium-chain saturated fatty acids

## Abstract

Persister cell formation and biofilms of pathogens are extensively involved in the development of chronic infectious diseases. Eradicating persister cells is challenging, owing to their tolerance to conventional antibiotics, which cannot kill cells in a metabolically dormant state. A high frequency of persisters in biofilms makes inactivating biofilm cells more difficult, because the biofilm matrix inhibits antibiotic penetration. Fatty acids may be promising candidates as antipersister or antibiofilm agents, because some fatty acids exhibit antimicrobial effects. We previously reported that fatty acid ethyl esters effectively inhibit *Escherichia coli* persister formation by regulating an antitoxin. In this study, we screened a fatty acid library consisting of 65 different fatty acid molecules for altered persister formation. We found that undecanoic acid, lauric acid, and N-tridecanoic acid inhibited *E. coli* BW25113 persister cell formation by 25-, 58-, and 44-fold, respectively. Similarly, these fatty acids repressed persisters of enterohemorrhagic *E. coli* EDL933. These fatty acids were all medium-chain saturated forms. Furthermore, the fatty acids repressed Enterohemorrhagic *E. coli* (EHEC) biofilm formation (for example, by 8-fold for lauric acid) without having antimicrobial activity. This study demonstrates that medium-chain saturated fatty acids can serve as antipersister and antibiofilm agents that may be applied to treat bacterial infections.

## Introduction

Persister cells are tolerant to conventional antibiotic treatment because they are metabolically dormant or grow slowly without acquiring inherent antibiotic resistance via genetic modifications [30], whereas resistant cells undergo genetic changes that block antibiotic activity [16]. Persister pathogens become active after the level of antibiotics decreases and thus cause chronic recalcitrant infections [19]. Persister cells are also highly tolerant to environmental stresses, such as low pH [25], nutrient starvation [4], hyperosmolarity [37], or heat shock [5]. Because of their high tolerance, persister cells are enriched in biofilms [21], sessile multi-microbial communities that are formed in response to environmental stresses [3] through secreted self-synthesized polymeric matrices [17]. Many bacteria, including pathogens, can form biofilms, but inhibiting biofilms is challenging because of poor antibiotic penetration, biofilm-specific gene regulation, and persister cell formation [35]. Hence, identifying new antimicrobial agents other than antibiotics is critical to decrease persister cell formation.

Fatty acid molecules, composed of a chain of carbon atoms with attached hydrogen atoms, are widely used in therapeutics, food preservation, and agriculture [12]. Some fatty acids inhibit or kill bacteria directly [51], whereas others affect virulence factors or prevent bacterial adhesion [44]. Fatty acids are produced in natural sources, such as plants that are inexpensive and non-toxic [43]. The antimicrobial activities of fatty acids depend on the structure, chain length, and degree of saturation [36]. As a result of these properties, fatty acids are considered as an alternative to conventional antibiotics for the treatment of infectious diseases. Furthermore, fatty acids exhibit antibiofilm properties in relation to antivirulence [28]. However, the roles of fatty acid molecules in altering persister cells have not been extensively studied. *cis*-2-decenoic acid produced from *Pseudomonas aeruginosa* was first identified for decreasing bacterial cells in a dormant state by causing them to transition to a metabolically active state via enhancing the expression of metabolic marker transcripts [32]. This compound has also been found to inhibit biofilm formation through enhancing biofilm dispersion [31]. Recently, we reported that ethyl esters of unsaturated fatty acids, including ethyl *trans*-2-decenoate, ethyl *trans*-2-octenoate, and ethyl *cis*-4-decenoate, repress *Escherichia coli* persister formation by regulating the antitoxin HipB [47]. Therefore, fatty acid molecules can be applied to control persister cell formation, thus preventing bacterial infections.

In this study, we screened 65 fatty acids with chain lengths of 10 to 24 carbons, including saturated and unsaturated forms, to determine whether combined antibiotic and fatty acid treatment might decrease persister cell formation. We identified antipersister and/or antibiofilm fatty acid molecules with the potential to be used with antibiotics to treat bacterial infections efficiently.

## Materials and Methods

### Bacterial Strains and Fatty Acids

*E. coli* BW25113 (*lacI*^q^ *rrnB*_T14_ Δ*lacZ*_WJ16_ *hsdR*514 Δ*araBAD*_AH33_ Δ*rhaBAD*_LD78_) [1], the *hipB* mutant (BW25113 Δ*hipB*) of the same strain, and enterohemorrhagic *E. coli* EDL933 (ATCC 43895) were used in this study. *E. coli* strains were cultured in 3 ml of LB medium at 220 rpm at 37°C overnight. The SCREEN-WELL Fatty Acid Library, purchased from Enzo Life Science (USA), contained 65 fatty acids (Table S1) dissolved in dimethyl sulfoxide (DMSO) with a stock concentration of 10 mM. Undecanoic acid (AC173970250), lauric acid (AC167281000), and N-tridecanoic acid (AC139520100) were purchased from Acros Organics (Morris Plains, USA) and are listed in Table 1.

### Persister Cell Formation Assays

Persister assays were conducted according to previously reported procedures [24]. Briefly, overnight *E. coli* cultures were diluted 100-fold in 25 ml of fresh LB medium in sterilized 250 ml flasks and incubated at 220 rpm at 37°C until an OD_600nm_ 0.7–0.9 was reached. Late stationary phase cells were harvested after overnight growth (18 h incubation following inoculation). Exponential phase or late stationary phase cells were harvested by centrifugation at 12,000 ×*g* for 5 min, washed twice with 0.85% NaCl solution, and adjusted to OD_600nm_ 1.0 with fresh LB. Then, the cells were exposed to each fatty acid at a final concentration of 0.1 or 1 mM in the presence of 5 μg/ml ciprofloxacin, 100 μg/ml ampicillin, or 100 μg/ml kanamycin for 3 h, 6 h, or 24 h at 220 rpm at 37°C. Cells were pelleted, washed twice with 0.85% NaCl solution, and diluted serially (10^1^–10^4^ dilution). Then 10 μl was applied to LB agar plates, which were incubated at 37°C. Persister cells were quantified by CFU counting.

### Biofilm Formation Assays

Biofilm formation assays were conducted according to a previously described method [15] with modifications. Briefly, overnight cultures were adjusted to an OD_600nm_ of 0.01 (approximately 5 × 10^6^ CFU/ml) with LB medium, and then 1.5 ml of adjusted culture was added to a polypropylene culture tube (Falcon PN352006, Corning, USA) with or without fatty acids. After incubation at 37°C for 24 h without shaking, the planktonic cultures were discarded, and the culture tubes containing biofilm were rinsed three times with 3 ml of 0.85% NaCl solution. Biofilm cells were carefully collected with a cotton swab [42] and resuspended in 3 ml of 0.85% NaCl solution via vortexing for 30 s. Biofilm cells were diluted serially (10^1^–10^6^ dilution), and 10 μl was applied on LB agar plates, which were incubated at 37°C. Biofilm cells were quantified as colony forming units (CFU) per surface area (cm^2^).

### Growth Measurement

Overnight cultures were adjusted to OD_600nm_ 0.05 with fresh LB medium, and then 200 μl cell suspensions were added to a 96-well plate. After the addition of different concentrations of fatty acids, cell growth was measured at OD_600nm_ every 20 min at 37°C on a Synergy HTX plate reader in fast shaking mode (Biotek, USA). Each data point was obtained from three replicate wells with two independent cultures.

### Statistical Analysis

Statistical analysis was performed with two-tailed Student’s *t*-test between ciprofloxacin only or DMSO control and each fatty acid treatment. The statistical significance is indicated in the figure with * (*p* < 0.001). All reported data are average ± standard deviation.

## Results and Discussion

### Identification of Undecanoic Acid, Lauric Acid, and N-Tridecanoic Acid as Inhibitors of *E. coli* BW25113 Persister Cell Formation

**Fig. 1:**
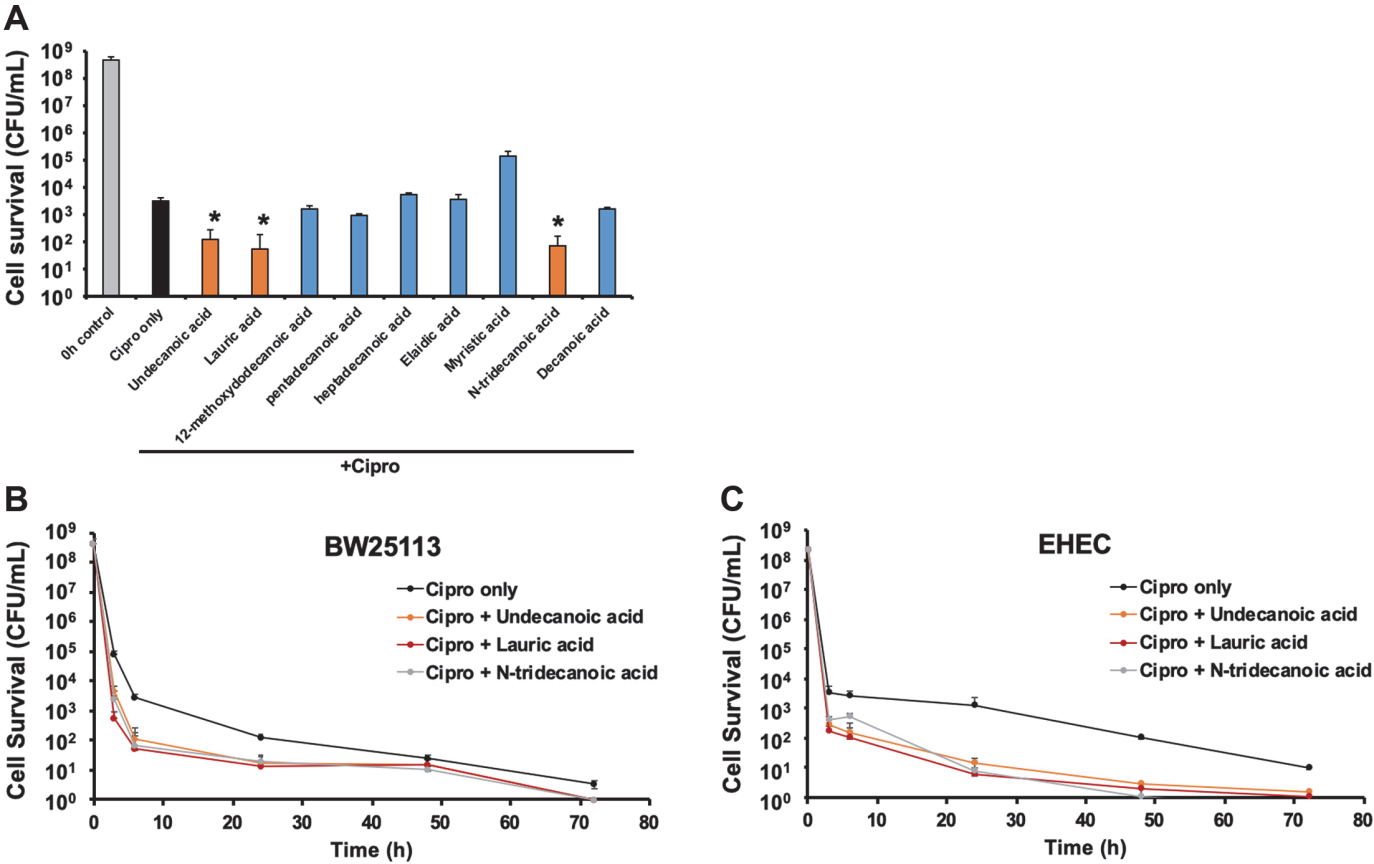
Fatty acid induced alterations in *E. coli* persister formation. (**A**) Exponentially grown *E. coli* BW25113 cells were exposed to 5 μg/ml of ciprofloxacin (Cipro) and 1 mM of nine fatty acids for 6 h in LB medium. (**B**) Exponentially grown BW25113 and (**C**) EDL933 (Enterohemorrhagic *E. coli*) cells were exposed to 5 μg/ml of ciprofloxacin and 1 mM of undecanoic acid, lauric acid, and N-tridecanoic acid for 3, 6, 24, 48, and 72 h in LB medium. Error bars indicate the standard deviation of two independent cultures with three replicates. *Indicates a significant difference relative to ciprofloxacin treatment only, with a *p*-value < 0.001.

To explore whether fatty acids can inhibit persister cell formation, we examined the ability of 65 fatty acids to alter persister cell formation. These fatty acids ranged in length from 10 to 24 carbons and were saturated or unsaturated. First, we treated exponential phase *E. coli* BW25113 cells with 5 μg/ml of ciprofloxacin and 0.1 mM of each fatty acid for 6 h at 37°C at 220 rpm and compared the levels of persister cells to those after ciprofloxacin only treatment. This screen resulted in the selection of nine fatty acids that decreased persister cells more than twofold. We then performed a second persister experiment with the nine candidate fatty acids by increasing their concentration to 1 mM to maximize their persister inhibition effect under the same incubation conditions (Fig. 1A). Undecanoic acid, lauric acid, and N-tridecanoic acid were identified as persister inhibitors that consistently decreased persister cell formation in the BW25113 strain, by 25-, 58-, and 44-fold, respectively.(Fig. 1A). Next, we examined persister cell formation at different time points. The incubation time was increased up to 72 h, and a biphasic cell survival curve [2] was obtained after ciprofloxacin treatment (Fig. 1B). Cotreatment with ciprofloxacin and each fatty acid further decreased the levels of persisters over the examination period, to levels below those after ciprofloxacin-only treatment. After longer time incubation, (24, 48, and 72 h), the cell survival after cotreatment was almost negligible (Fig. 1B). These results indicate that undecanoic acid, lauric acid, and N-tridecanoic acid repress persister formation in the BW25113 strain.

Interestingly, the three fatty acids obtained from the persister inhibition screening were all saturated fatty acids of medium-chain length (undecanoic acid, 11 carbon atoms (C11); lauric acid, C12; and N-tridecanoic acid, C13)(Table 1), whereas a collection of various-length (C10 to C24) fatty acids of saturated and unsaturated states were studied (Table S1). *cis*-2-decenoic acid, which is a known as antipersister and antibiofilm fatty acid, is medium-chain (C10) and unsaturated [32]. Previously, we investigated nine fatty acids and their derivatives [47] that were unsaturated and structurally similar to *cis*-2-decenoic acid and found that medium-chain fatty acid derivatives containing ethyl esters (ethyl *trans*-2-decenoate, ethyl *trans*-2-octenoate, and ethyl *cis*-4-decenoate) inhibit persister formation [47]. Longer-chain fatty acids with greater than C14, such as pentadecanoic acid, heptadecanoic acid, eliadic acid, and myristic acid (Fig. 1A), shorter-chain fatty acid such as decanoic acid (Fig. 1A), and the fatty acid derivatives with C6 to C9 [47] were not able to decrease persister level. Although cellular responses depend on specific fatty acid structural characters, including length, saturation level, and type and location of functional groups [33], nonetheless the chain length of fatty acids may be a critical consideration with respect to antipersister activity, according to previous [32, 47] and current studies. Regulation of bacterial toxin-antitoxin systems [47], increasing protein synthesis [32], or influencing membrane fluidity [28] by fatty acids might cause cells to be more susceptible to antibiotic treatment. However, undecanoic acid, lauric acid, and N-tridecanoic acid in this study were all saturated forms distinct from the previously reported antipersister fatty acid compounds, thus suggesting that they may have a different mechanism of inhibiting bacterial persisters. Further systematic studies will be necessary to understand the specific interactions of medium-chain saturated fatty acids with antibiotics or bacteria during persister formation.

### Undecanoic Acid, Lauric Acid, and N-Tridecanoic Acid Inhibit Enterohemorrhagic *E. coli* (EHEC) Persister Formation

To investigate the effects of these fatty acids on other *E. coli* strains, we examined the time course of persister formation of the EHEC strain, a pathogenic *E. coli* responsible for outbreaks of bloody diarrhea and hemolytic uremic syndrome, and a major source of food poisoning [38]. The persister level of EHEC in the presence of ciprofloxacin 5 μg/ml was 0.0005% (2.5 × 10^3^ CFU/ml) after a 3 h incubation (Fig. 1C), a value 20-fold lower than that of BW25113 (Fig. 1B). This result was consistent with our previous observation of EHEC persister cell formation [47]. When we added 1 mM of undecanoic acid, lauric acid, and N-tridecanoic acid during EHEC persister formation for 3 h, the persister level was 11-, 21-, and 9-fold lower, respectively, than that after ciprofloxacin-only treatment (Fig. 1C). Moreover, extended incubation (up to 72 h) with each fatty acid and ciprofloxacin resulted in nearly complete inactivation of EHEC cells (Fig. 1C). Therefore, undecanoic acid, lauric acid, and N-tridecanoic acid effectively inhibit persister formation of *E. coli*.

**Fig. 2:**
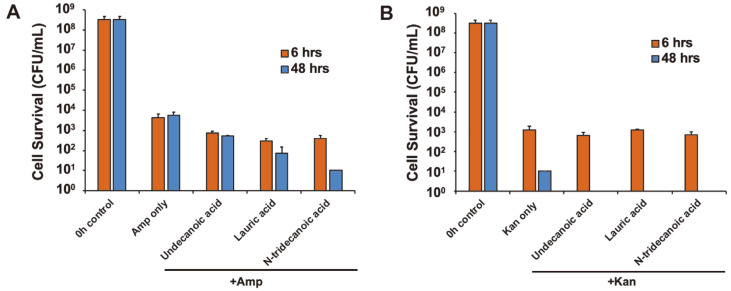
EHEC persister cell formation in the presence of different antibiotics. EHEC persister formation with 100 μg/ml of (**A**) ampicillin (Amp) and (**B**) kanamycin (Kan) together with 1 mM of fatty acids for 6 and 48 h in LB. Error bars indicate the standard deviation of two independent cultures with three replicates. *Indicates a significant difference relative to Cipro treatment only, with a *p*-value < 0.001.

Typically, antibiotics repress bacterial growth through different inhibition mechanisms. For example, ciprofloxacin (fluoroquinolone) inhibits DNA replication and repair, ampicillin (β-lactam) blocks cell-wall synthesis, and kanamycin (aminoglycosides) represses protein synthesis [14]. Therefore, we investigated the effects of the medium-chain saturated fatty acids during EHEC persister formation in the presence of different classes of antibiotics in addition to ciprofloxacin. Specifically, we examined EHEC persister formation with 100 μg/ml of ampicillin (Fig. 2A) or kanamycin (Fig. 2B) in the presence of 1 mM of each fatty acid for 6 and 48 h. Similarly to the results with ciprofloxacin and each fatty acid cotreatment (Fig. 1B), the addition of undecanoic acid, lauric acid, and N-tridecanoic acid repressed persister cell formation caused by ampicillin treatment (6-, 14-, and 10-fold lower, respectively, than that after ampicillin-only treatment after 6 h; Fig. 2A). However, we did not observe a noticeable difference during kanamycin treatment with or without fatty acids (Fig. 2B). Cell survival after 48 h of kanamycin exposure (10 CFU/ml) was considered as negligible as it was close to the complete cell killing with the cotreatment (Fig. 2B). These results suggest that the fatty acid molecules that we tested behave differently when persister cells are formed through various bacterial inactivation mechanisms in the presence of different antibiotics.

Persister cell formation is known to be an antibiotic-specific response rather than a global metabolic dormancy [20]. The SOS response after DNA damage by ciprofloxacin induces toxin-antitoxin (TA) transcripts in *E. coli*, thus resulting in persister formation [50], and ampicillin treatment induces TA pairs, thereby increasing persister cells [40]. However, motility and amino acid biosynthesis play important roles in gentamycin (aminoglycoside)-induced persister cell formation [41]. We previously reported that unsaturated fatty acid ethyl esters with ciprofloxacin increase the antitoxin HipB, thus decreasing persister formation, and similar persister inhibition was achieved after cotreatment with ampicillin [47]. We performed persister assay of *E. coli* BW25113 Δ*hipB* strain in the presence of ciprofloxacin and each fatty acid. Corroborating to the previous study [47], the persister level of *hipB* mutant upon ciprofloxacin was 100-fold higher (Fig. S1) that that of the wild-type BW25113 strain (Fig. 1B). Persister formation of *hipB* mutant was decreased by the cotreatment of fatty acid and ciprofloxacin in comparison to the ciprofloxacin-only treatment after 6 and 48 h incubation (Fig. S1), which performed similar trend to the persister formation of wild-type BW25113 strain by the cotreatment (Fig. 1B). Hence, the decrease of persister cell formation by undecanoic acid, lauric acid, and N-tridecanoic acid was not associated with the work of antitoxin HipB. However, because *E. coli* possesses multiple TA systems [45], the possibility remains that undecanoic acid, lauric acid, and N-tridecanoic acid might influence TA-related cellular regulation and consequently could enhance persister cell control.

We determined that fatty acids did not repress persister formation induced by kanamycin (Fig. 2B). Kanamycin binds the 30S ribosomal subunit and inhibits protein synthesis [6]; as a result, ribosome dimerization induced by the presence of aminoglycosides, including kanamycin, results in the ribosome hibernation and thereby increases bacterial tolerance [34]. Recently, the alarmone guanosine pentaphosphate/tetraphosphate (ppGpp) ribosome dimerization persister model has been proposed to understand how cells enter and exit the persister state [46, 48]. Persister cells are directly formed by inactivating ribosomes via ppGpp with the evidence that persister cells contain a large fraction of 100S ribosomes [46]. Therefore, it is possible that the fatty acids might interact with ribosomes to interrupt the pathway of persistence formation, whereas kanamycin-induced persister cells containing inactivated ribosomes might not be affected by the fatty acids.

### Persister Formation in Stationary Phase Cells Is Not Affected by Fatty Acids

To investigate the effect of fatty acids on stationary phase persister cell formation, we directly harvested the overnight culture (18 h after inoculation) and adjusted its optical density to 1.0 with fresh LB media in the presence of 1 mM of each fatty acid and 5 μg/ml of ciprofloxacin. The population of persister cells in the stationary phase with ciprofloxacin-only treatment was increased by more than 10-fold in comparison to that in the exponential phase. However, unlike the exponential phase of persister cell formation, the effect of fatty acid inhibition on persister cell formation was diminished in the stationary phase cells of BW25113 and EHEC (Fig. S2). Stationary phase cells have more robust membranes with a rigid cell envelope, a highly cross-linked cell wall, and a reduced membrane fluidity in comparison to exponential phase cells, which exhibit a high membrane fluidity [22]. Bacteria can incorporate free exogenous fatty acids from its environment to their cellular membrane [13], such that the incorporation of fatty acids into the membrane may increase membrane fluidity and thereby enhance antimicrobial susceptibility [11, 28]. Our results that fatty acids are effective toward decreasing the persister formation of exponential phase cells but are less effective in regard to stationary phase cells, implying that undecanoic acid, lauric acid, and N-tridecanoic acid might increase membrane fluidity and improve the ciprofloxacin efficacy for inhibiting persister formation. It remains necessary to elucidate the relation between the cell membrane fluidity, persister formation, and the effect of fatty acids on membrane fluidity.

### Undecanoic Acid, Lauric Acid, and N-Tridecanoic Acid Do Not Exhibit Antimicrobial Activity against EHEC Cells

**Fig. 3:**
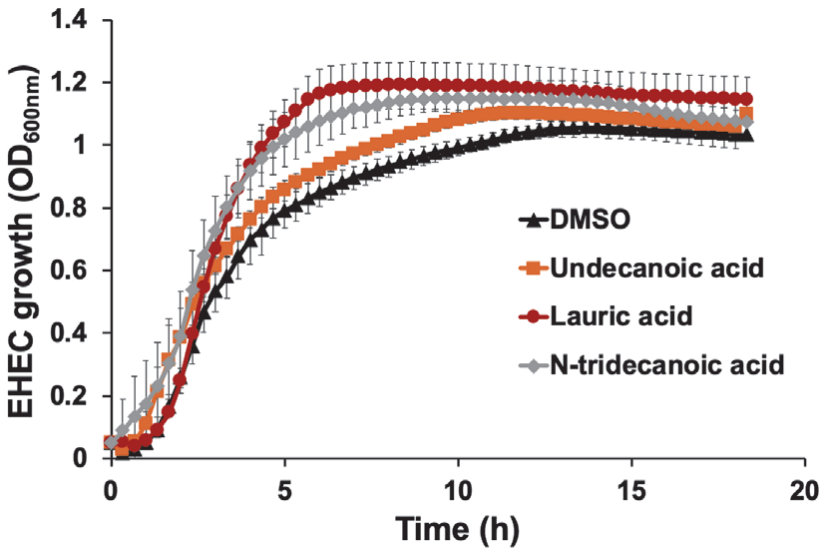
EHEC growth with undecanoic acid, lauric acid, and N-tridecanoic acid. The growth of EHEC culture was monitored until 18 h in the presence of 1 mM of each fatty acid at 37°C in LB. Cell growth at OD_600nm_ was measured every 20 min. Error bars indicate standard deviations of two independent cultures with three replicates.

Some fatty acids, such as linolenic acid, myristic acid, and lauric acid, have antimicrobial activity against microorganisms such as *Bacillus subtilis*, *Listeria monocytogenes*, *Mycobacterium smegmatis*, and *Staphylococcus aureus* [7]. Because the identified fatty acids (undecanoic acid, lauric acid, and N-tridecanoic acid) effectively inhibited persister cell formation (Fig. 1), we investigated whether their inhibition effects might be derived from their antimicrobial activity. Specifically, we monitored the growth of EHEC cells in the presence of 1 mM of undecanoic acid, lauric acid, or N-tridecanoic acid (Fig. 3). The final cell growth OD in the late stationary growth phase after 15 h was similar among the DMSO control and fatty acid-treated samples with no significant difference (Fig. 3), indicating that undecanoic acid, lauric acid, and N-tridecanoic acid do not exhibit antimicrobial activity against EHEC cells. However, there was a slight improvement in cell growth with fatty acids, particularly during 3 to 13 h of incubation (Fig. 3). Reportedly, many fatty acids exhibit antimicrobial activity in a selective manner [7, 28, 51]. On the one hand, fatty acids inhibit Gram-positive bacteria [7, 10, 12]. On the other hand, fatty acids are metabolized in bacteria to maintain lipid homeostasis as well as for use as carbon and energy sources [18, 23]. Therefore, EHEC cells might utilize fatty acids to enhance cellular growth during the exponential and early stationary growth phases. Previous studies also suggest that the antipersister activity of fatty acid molecules is not derived from the antimicrobial activity but is based on specific intracellular interactions affecting protein synthesis or regulation [32, 47]. Fatty acid molecules may not induce bacterial burden (Fig. 3) but may synergistically increase the efficacy of antibiotics in killing bacteria (Fig. 1). Persister cell inhibition without direct antimicrobial activity might be an attractive approach, because such antipersister compounds may mitigate the development of antimicrobial resistance of bacteria by maximizing the function of conventional antibiotics in inactivating bacteria.

### Fatty Acids Repress EHEC Biofilm Formation

**Fig. 4:**
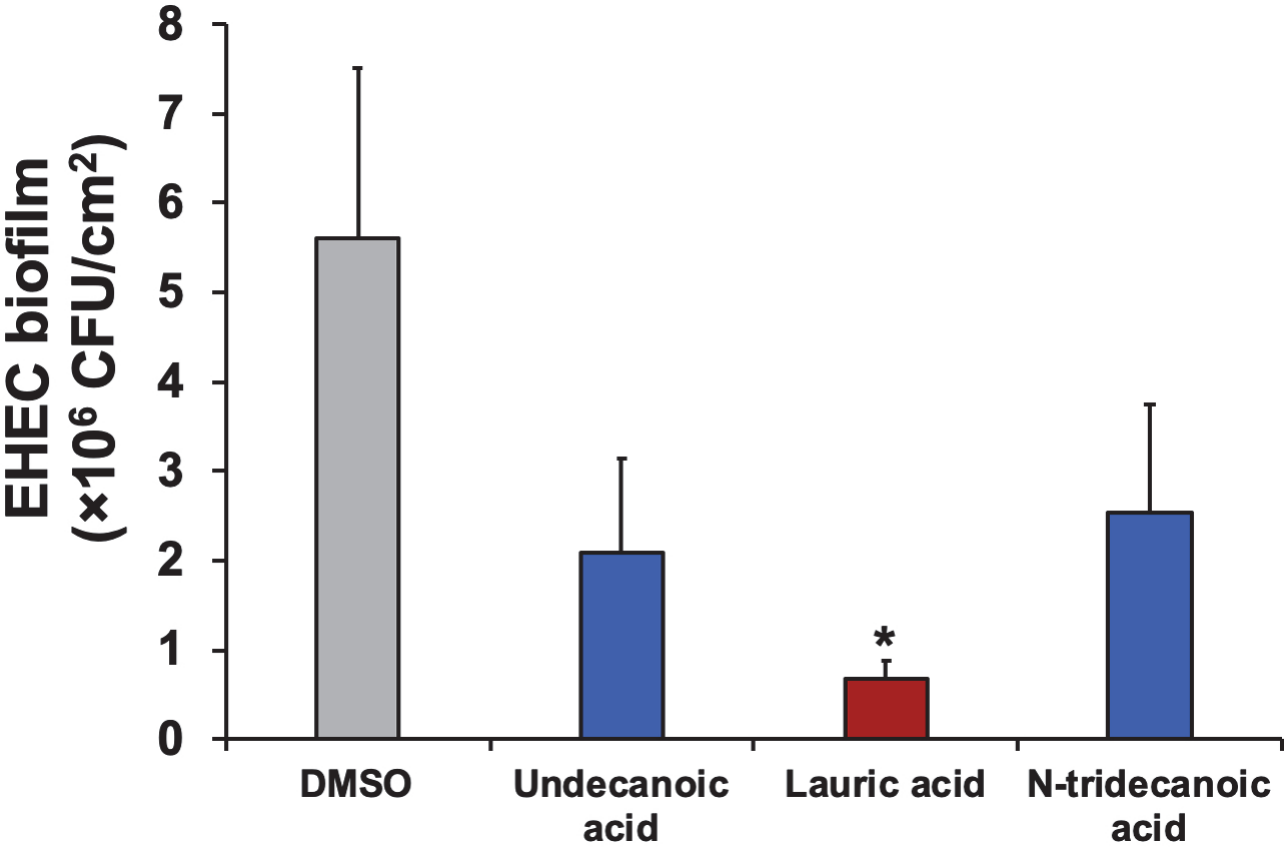
EHEC biofilm formation. EHEC biofilms were formed in the presence of 1 mM of undecanoic acid, lauric acid, and N-tridecanoic acid for 24 h at 37°C without shaking. Error bars indicate standard deviations of two independent cultures with three replicates. *Indicates a significant difference relative to the DMSO control, with a *p*-value < 0.001.

High persister populations in biofilms enhance the tolerance of biofilm cells [8, 39]. Because the examined fatty acid molecules inhibited persister cell formation, we reasoned that these fatty acids might be applied for repressing biofilm formation. We exposed EHEC cells to undecanoic acid, lauric acid, and N-tridecanoic acid during 24 h of biofilm formation. As expected, all fatty acids tested repressed EHEC biofilms, and lauric acid exhibited the highest efficacy (8-fold; Fig. 4). Lauric acid inhibits *Clostridium difficile* biofilm formation and disrupts preformed biofilms through the antimicrobial activity of lauric acid [49]. Recently, we reported that the combined treatment of lauric acid and the antimicrobial peptide nisin represses the development of nisin resistance of *L. monocytogenes* and inactivates cells in biofilms [52]. However, our results indicated that without having antimicrobial activity toward EHEC (Fig. 3), lauric acid inhibits EHEC biofilm formation (Fig. 4). Fatty acids can serve as signaling molecules in cell-cell communication, e.g., diffusible signal factor [27]. A previous study has indicated that *cis*-2-decenoic acid functions as a signaling molecule that stimulates the biofilm dispersion response and thus inhibits biofilm formation by Gram-positive and Gram-negative bacteria [9, 31]. However, these fatty acid signaling molecules are typically short-chain unsaturated fatty acids with carbon 2 in a *cis* configuration [31]. Fatty acids also can regulate virulence factors and prevent bacterial adhesion [9]. For example, some saturated fatty acids, such as lauric acid (12 carbon atoms), myristic acid (14 carbon atoms), and palmitic acid (16 carbon atoms), inhibit *Proteus mirabilis* biofilm formation by regulating swarming and virulence factor expression [29]. Various fatty acids possess ability to inhibit biofilm development by affecting the adhering surface and cell membrane fluidity [28]. For example, linoleic acid inhibits *P. aeruginosa* biofilms by increasing the membrane permeability of the bacteria [26]. Given the close relationships observed among persister cells, biofilms, and virulence factors, as well as our observations that undecanoic acid, lauric acid, and N-tridecanoic acid exhibited antipersister and antibiofilm activities, medium-chain saturated fatty acids may play important roles by connecting persister cell repression, biofilm inhibition, and potentially virulence regulation.

This study demonstrates that the three fatty acid molecules undecanoic acid, lauric acid, and N-tridecanoic acid effectively inhibit *E. coli* persistence as well as biofilm formation. Fatty acids are non-toxic, naturally produced products that are safe to humans [43] and have promise for applications in therapeutics, food preservation, and agriculture [12]. Therefore, medium-chain saturated fatty acids may serve as new antipersister and/or antibiofilm agents to combat antibiotic-resistant bacterial infections.

## Supplemental Material

Supplementary data for this paper are available on-line only at http://jmb.or.kr.

## Figures and Tables

**Table 1:** List of three selected fatty acids.

Fatty acid	Structure	Chemical formula	Molecular weight
Undecanoic acid	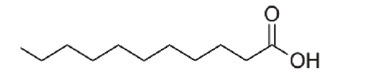	C_11_H_22_O_2_	186.29
Lauric acid	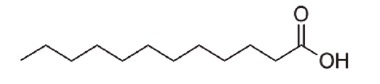	C_12_H_24_O_2_	200.32
N-tridecanoic acid	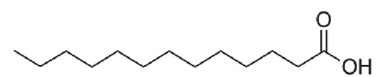	C_13_H_26_O_2_	214.35

Undecanoic acid, lauric acid, and N-tridecanoic acid were selected as antipersister molecules from BW25113 persister cell screening.
