# Supplementary material for: Undecanoic Acid, Lauric Acid, and N-Tridecanoic Acid Inhibit Escherichia coli Persistence and Biofilm Formation
Source: J Microbiol Biotechnol. 2020 Oct 13;31(1):130–6. doi: 10.4014/jmb.2008.08027 (PMC8513074; doi:10.4014/jmb.2008.08027)
Supplement: Supplementary file 1 [file jmb-31-1-130-supple.pdf]

J. Microbiol. Biotechnol.
https://doi.org/10.4014/jmb.2008.08027

Supplementary Table S1. List of 65 fatty acids from this study. Nine fatty acids indicated as **bold** were selected for further screening.

No.	Name	MW	CAS
1	**Decanoic acid**	172.3	334-48-5
2	**Undecanoic acid**	186.3	112-37-8
3	10-Undecenoic acid	184.3	112-38-9
4	**Lauric acid (Dodecanoic acid)**	200.3	143-07-7
5	11-Dodecenoic acid	198.3	65423-25-8
6	**N-tridecanoic acid (tridecanoic acid)**	214.3	638-53-9
7	**12-Methoxydodecanoic acid**	230.3	92169-28-3
8	12-Tridecenoic acid	212.3	6006-06-0
9	**Myristic acid (Tetradecanoic acid)**	228.4	544-63-8
10	9(Z)-Tetradecenoic acid	226.4	544-64-9
11	9(E)-Tetradecenoic acid	226.4	544-64-9
12	**Pentadecanoic acid**	242.4	1002-84-2
13	10(Z)-Pentadecenoic acid	240.4	84743-29-3
14	10(E)-Pentadecenoic acid	240.4	321744-58-5
15	Hexadecanoic acid	256.4	57-10-3
16	9(Z)-Hexadecenoic acid	254.4	373-49-9
17	9(E)-Hexadecenoic acid	254.4	10030-73-6
18	**Heptadecanoic acid**	270.5	506-12-7
19	10(Z)-Heptadecenoic acid	268.4	29743-97-3
20	10(E)-Heptadecenoic acid	268.4	126761-43-1
21	Octadecanoic acid	284.5	57-11-4
22	6(Z)-Octadecenoic acid	282.5	593-39-5
23	6(E)-Octadecenoic acid	282.5	593-40-8
24	9(Z)-Octadecenoic acid	282.5	112-80-1
25	**Eliadic acid (9(E)-Octadecenoic acid)**	282.5	112-79-8
26	11(Z)-Octadecenoic acid	282.5	506-17-2
27	11(E)-Octadecenoic acid	282.5	693-72-1
28	9(Z), 11(Z)-octadecadienoic acid	280.4	60-33-3
29	(9Z,11E)-Octadecadienoic acid	280.4	2540-56-9
30	9(E),12(E)-Octadecadienoic acid	280.4	506-21-8
31	9(Z), 12(Z), 15(Z)-Octadecatrienoic acid	278.4	463-40-1
32	6(Z), 9(Z), 12(Z)-Octadecatrienoic acid	278.4	506-26-3
33	Nonadecanoic acid	298.5	646-30-0
34	7(Z)-Nonadecenoic acid	296.5	118020-79-4
35	7(E)-Nonadecenoic acid	296.5	191544-99-7
36	10(Z)-Nonadecenoic acid	296.5	73033-09-7
37	10(E)-Nonadecenoic acid	296.5	147527-21-7

38	10(Z),13(Z)-Nonadecadienoic acid	294.5	29204-20-4
39	6(Z), 9(Z), 12(Z), 15(Z)-Octadecatetraenoic acid	276.4	115610-41-8
40	Eicosanoic acid	312.5	506-30-9
41	11(E)-Eicosenoic acid	310.5	62322-84-3
42	8(Z)-Eicosenoic acid	310.5	76261-96-6
43	11(Z)-Eicosenoic acid	310.5	5561-99-9
44	5(Z)-Eicosenoic acid	310.5	7050-07-9
45	5(Z), 8(Z)-Eicosadienoic acid	308.5	125535-07-1
46	5(Z),8(Z)-7Dimethyleicosadienoic acid	336.6	89560-01-0
47	11(Z), 14(Z)-Eicosadienoic acid	308.5	2091-39-6
48	5(Z), 8(Z), 11(Z)-Eicosatrienoic acid	306.5	20590-32-3
49	8(Z), 11(Z), 14(Z)-Eicosatrienoic acid	306.5	1783-84-2
50	11(Z), 14(Z), 17(Z)-Eicosatrienoic acid	306.5	2091-27-2
51	5(Z), 8(Z), 11(Z), 14(Z)-Eicosatetraenoic acid	304.5	506-32-1
52	5(Z), 8(Z), 11(Z), 14(Z), 17(Z)-Eicosapentaenoic acid	302.5	10417-94-4
53	Heneicosanoic acid	326.6	2363-71-5
54	12(Z) Heneicosenoic acid	324.5	3515-84-2
55	13(Z)-Docosenoic acid	338.6	112-86-7
56	13(E)-Docosenoic acid	338.6	506-33-2
57	13(Z),16(Z)-Docosadienoic acid	336.6	17735-98-7
58	13(Z), 16(Z), 19(Z)-Docosatrienoic acid	334.5	28845-86-5
59	7(Z), 10(Z), 13(Z), 16(Z)-Ocosatetraenoic acid	332.5	28874-58-0
60	7(Z), 10(Z), 13(Z), 16(Z), 19(Z)-Docosapentaenoic acid	330.5	2234-74-4
61	4(Z), 7(Z), 10(Z), 13(Z), 16(Z), 19(Z)-Docosahexaenoic acid	328.5	6217-54-5
62	14(Z)-Tricosenoic acid	352.6	105305-008
63	14(E)-Tricosenoic acid	352.6	164414-85-1
64	Tetracosanoic acid	368.6	557-59-5
65	15(Z)-Tetracosenoic acid	366.6	506-37-6

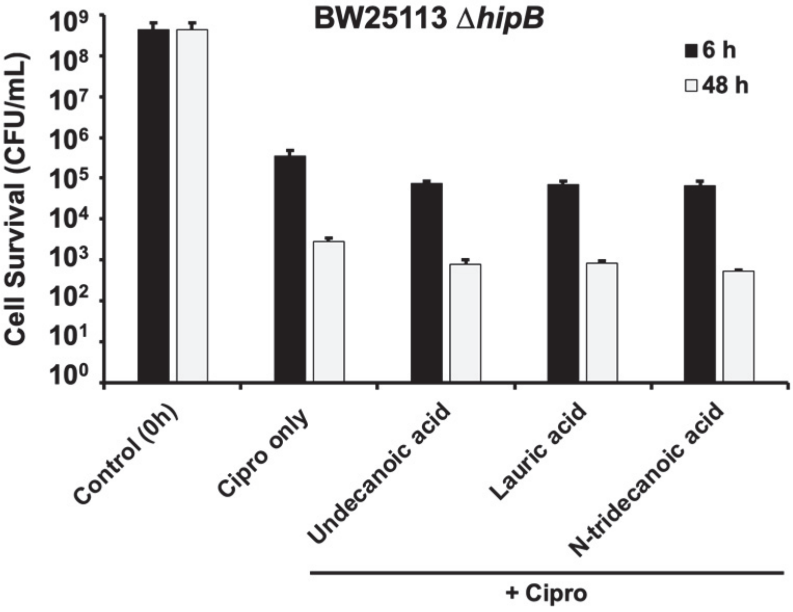

Supplementary Figure S1. Persister formation of *hipB* mutant. Exponentially grown BW25113 Δ*hipB* cells were exposed to 5 μg/ml of ciprofloxacin and 1 mM of undecanoic acid, lauric acid, and N-tridecanoic acid for 6 and 48 h in LB medium. Error bars indicate the standard deviation of two independent cultures with three replicates.

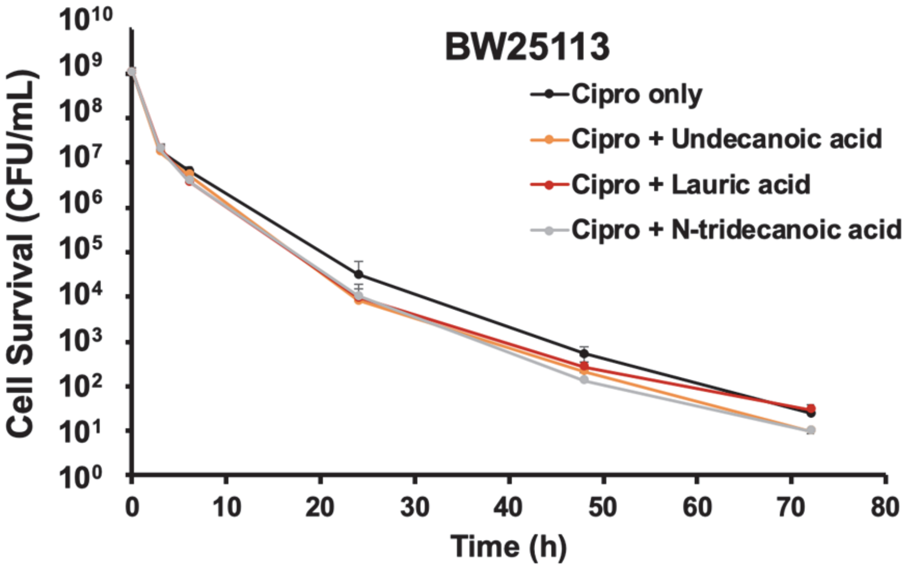

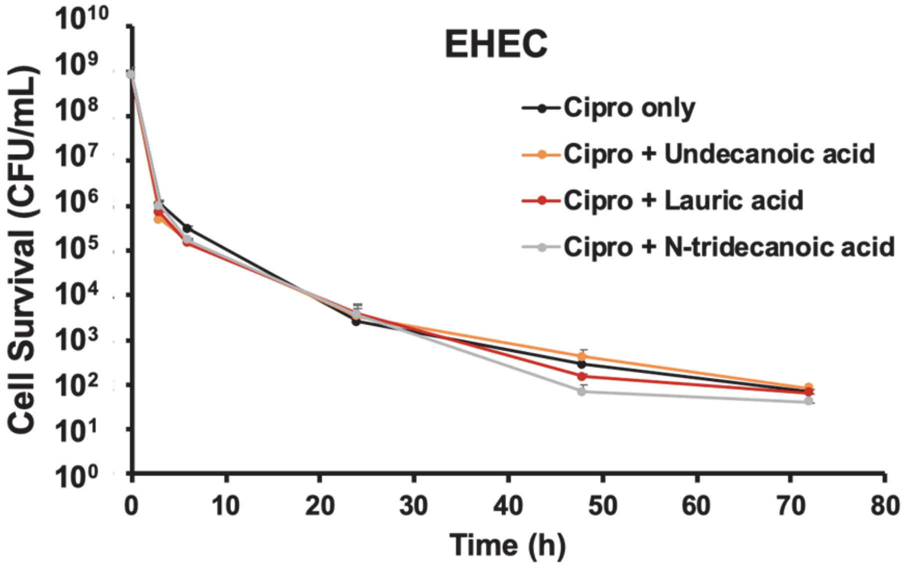

Supplementary Figure S2. Stationary phase persister cell formation. Overnight *E. coli* BW25113 and EHEC cells were exposed to 5 μg/ml of ciprofloxacin and 1 mM of undecanoic acid, lauric acid, and N-tridecanoic acid for 3, 6, 24, 48, and 72 h in LB medium. Error bars indicate the standard deviation of two independent cultures with three replicates.
